# Chinese caregivers’ experiences parenting children with autism spectrum disorder: a descriptive qualitative study

**DOI:** 10.3389/fpsyt.2025.1514948

**Published:** 2025-06-03

**Authors:** Xing Fan, Kyung Soon Ko

**Affiliations:** ^1^ College of Preschool Education, Capital Normal University, Beijing, China; ^2^ Dept. Creative Arts Psychotherapy, Jeonju University, Jeonju, Republic of Korea

**Keywords:** autism spectrum disorder, caregivers, China, children, parenting experiences

## Abstract

**Background:**

This study explored the parenting experiences of caregivers that are raising children with autism spectrum disorder in China by focusing on their emotional experiences as well as perceived roles and responsibilities.

**Objects:**

The research questions were (1) What are Chinese caregivers’ emotional experiences with their child with autism spectrum disorder? and (2) How do Chinese caregivers perceive their roles and responsibilities related to their children with autism spectrum disorder?

**Method:**

Qualitative data were gathered through individual interviews with caregivers of children with autism spectrum disorder. The MAXQDA software program was used for data analysis.

**Result:**

The data analysis resulted in four final themes: (a) Unable to abandon the hope that the child will become “ordinary,” (b) The child’s development goes against the expectations of their caregivers, (c) Emotional phases in the caregiver’s responses to diagnosis, and (d) Enduring the life that was given.

**Conclusion:**

This study sheds light on the experience of Chinese parents raising children with autism spectrum disorder in a culture that might perceive this disorder differently than the West. This study reflects the authentic parenting dilemmas and intense emotional fluctuations experienced by Chinese caregivers of children with autism spectrum disorder. Understanding these experiences will stimulate the readers’ humanistic thinking, expand their care and compassion toward this group, and benefit society. By exploring the emotional struggles and parenting challenges faced by these parents, the goal is to offer them more tangible understanding and social support.

## 1 Introduction

Autism spectrum disorder (ASD) is a behaviorally defined complex neurodevelopmental disorder, commonly diagnosed in early childhood (1). Its core symptoms include social interactional deficits and repetitive and limited behavioral patterns or interests (2). At present, the prevalence of ASD in China cannot be confirmed owing to the lack of a national-level monitoring system and epidemiological investigation (3, 4). According to the latest Chinese population census statistics, over 300 million Chinese children are under the age of 14. Conservatively estimated based on ASD’s global prevalence rate of 1% (5), approximately 3–5 million children in China have ASD (6, 7).

The parenting and emotional experiences of these children’s parents have also become undeniably difficult owing to the increasing incidence of ASD. A literature review found that existing studies have primarily focused on children with ASD or the effectiveness of therapeutic interventions for them (8–10). The research on caregivers of children with autism primarily focuses on parenting stress (3, 11, 12), social support needs (11), and the effects of stigmatization (13–15). However, research that delves deeply into the experiences of caregivers based on Chinese cultural characteristics is still lacking. The role and experience of parents are crucial in influencing the growth and development of children, whether typical or with special needs.

Under simultaneous and interactive effects of multiple factors, parents of children with ASD are known to experience higher levels of pressure than those of children without ASD (11, 16–18). Additionally, Chinese parents are influenced by traditional family values, and mothers bear more parenting responsibilities (19). Mothers also make more sacrifices, such as giving up opportunities for employment to take care of their children with ASD full-time (20).

In addition to the above factors, the severity of ASD symptoms and challenging behaviors in children are greater sources of stress for these parents. Longitudinal studies have shown that emotional and behavioral problems in children with ASD can persist for several years (21, 22), which further frustrates parents who find it challenging to manage them. Parents are often accused of “incompetence in discipline” owing to their children’s abnormal behavior (20). Therefore, these parents are forced to isolate themselves from society (23) to reduce and avoid instances wherein their children create a scene in public (13, 24).

### 1.1 Stigma against children with ASD and their caregivers

Goffman (1963) defined *stigma* as “a phenomenon whereby an individual with an attribute which is deeply discredited by his/her society is rejected because of the attribute (25). *Shaming* is a process through which an individual’s normal identity is disrupted due to others’ reactions, such as doubting, denying, and labeling” (p. 3). Owing to limited social and public understanding of ASD, parents of children with ASD are subjected to various forms of stigma and social hostility (26, 27). For example, in an educational environment, when children with ASD exhibit problematic and antisocial behaviors, such as getting angry, biting objects, and showing aggression, their parents are often accused of being unreasonable and not providing adequate education for their children (28, 29). When children with ASD exhibit unusual social behaviors, such as creating loud noises, spitting, or showing impulsive behavior in public, parents are often criticized and ridiculed by others (30). A survey by Hua and Yang (29) showed that 70% of parents of children with ASD reported being discriminated against or ignored by society. Several studies have confirmed that parents can feel embarrassed by the problematic behavior of their children with ASD and reduce or stop going out with them (13, 24, 31).

In Chinese culture, parents are greatly influenced by the cultural concepts of Confucianism, Buddhism, and Taoism. They bear more psychological burdens compared to parents from other cultures, due to the significance of reputation and individual social identity in the collectivist culture (14, 27, 32). Chinese cultural beliefs regarding *karma* and cause and effect can lead a family to feel responsible when their child is diagnosed with ASD (15, 33). *Karma* refers to the concept of cause and effect in Chinese culture. It is believed that a close moral connection exists between cause and effect; doing good leads to receiving good, and doing evil leads to receiving evil (34). Caregivers will first doubt and blame themselves, believing their moral “flaws” have led to such retribution (35). Extended families also feel a sense of burden when a member is diagnosed with ASD (14, 36).

Then, caregivers will view children with ASD as “bad seeds” and a stain on the family (37) and hide their children or deliberately conceal their actual situation (38, 39). Additionally, Chinese parents usually seek medical attention repeatedly after learning of their children’s ASD diagnosis. They hope doctors can provide a “lighter diagnosis” like “developmental delay” (39). The above reactions from Chinese parents are their response to disease stigma and specific social and cultural factors (32).

In the West, individualism and cultural diversity are generally advocated. The core values of individualism emphasize placing individual needs above group needs (40), starkly contrasting the collectivist values advocated in China. The cultural concepts of diversity and individualism also influence society’s perception of and attitude toward individual differences. The neurodiverse movement is a typical manifestation of this cultural belief. It actively advocates for understanding and accepting the phenomenon of neurodiversity, including its manifestation in autism (41).

In addition, in the West, the role of caregivers when caring for a disabled child is also different from the role of caregivers in China. Western caregivers often participate more actively in the child’s treatment process, which helps to build a cooperative relationship with the medical team based on mutual trust, respect, and honesty (42–44). The role of mothers is particularly prominent, as they are seen as key drivers of their children’s future development (45). These mothers are committed to searching for the best intervention measures and related services for their children to maximize their potential, rather than delving into the origins of karma and retribution or avoiding their children’s challenges.

In summary, there are significant differences in the reactions and attitudes of caregivers of children with ASD between China and the West. In the context of Chinese culture, caregivers are often deeply influenced by the concept of “saving face” and group identification and, therefore, tend to adopt conservative or covert strategies in dealing with their children’s disability (14, 27, 38). In the West, caregivers emphasize individual development and have a more proactive attitude toward coping. They seek professional support actively and strive for effective intervention measures through open discussions (46). These differences reflect people’s understanding of family roles in different cultural backgrounds.

Given the impact of ASD on children and their parents, its increasing prevalence, and the fact that autism is a lifelong disorder, understanding the parenting and emotional experiences of these parents can be crucial. However, existing research has not sufficiently explored the parenting experiences of Chinese children with ASD within cultural contexts.

This study aims to shed light on the experience of Chinese parents raising children with ASD in a culture that might perceive ASD differently than the West. The overarching research question is: what is the caregiver’s experience of parenting a child with ASD in China?

Research Question 1: What are Chinese caregivers’ emotional experiences of raising their children with ASD?

Research Question 2: How do Chinese caregivers perceive their roles and responsibilities related to their children with ASD?

## 2 Method

This study adopted a descriptive qualitative research method and intended to explore the parenting experiences of parents raising children with ASD in China. A descriptive, qualitative research method is suitable for studies that do not require an in-depth theoretical background, especially for fields with limited knowledge of the study’s topic. This method approaches, observes, and directly describes participants’ experiences (47). This design’s advantage lies in recognizing the problem’s subjective nature, the participants’ different experiences, and its ability to directly reflect or use terms most similar to those used in the initial research question when presenting results (48, 49).

### 2.1 Recruitment of participants

Through purposeful sampling, we have recruited participants who can provide the most information for this study. Owing to the constraints of specific Chinese cultural concepts, caregivers often view their children’s ASD and problematic behaviors as their own or their family’s fault and remain vigilant and resistant to the potential exposure of these “disgraceful family matters” (38). In this limited context, five participants were recruited from a clinical center where their children receive dance/movement therapy (DMT). Their children have received or continue to receive one-on-one DMT services from the researcher.

Based on the long-standing and stable therapeutic relationship between the researcher and the child, the researcher maintained regular interactions with caregivers, such as post-session parent communications and periodic parental consultations. As the therapeutic relationship deepened, mutual trust developed between the researcher and caregivers. This trusting dynamic ensured profound depth in interviews, enabling participants to authentically express their inner thoughts and emotional experiences. For instance, during interviews, participants openly allowed the researcher to explore their parental journeys, personal reflections, and familial dynamics, reflecting nuanced dimensions of their lived realities.

All families resided in China. Recruitment criteria were (1) caregivers of children diagnosed with ASD by the pediatric psychiatric department of certified hospitals (2), parents or caregivers who play a significant role in caring for their children with ASD, and (3) parents or caregivers willing to share their experiences of parenting children with ASD.

### 2.2 Description of the participants

The five participants (three fathers and two mothers) were between 35 and 50 years old.

One family had two daughters, and all the other families have only one child. These children were diagnosed with ASD around the age of three. DSM-5 categorizes ASD symptoms into two main categories: deficits in social communication and social interaction, and restricted, repetitive patterns of behavior, interests, or activities. According to this classification framework, the diagnosis of ASD requires individuals to exhibit persistent deficits in all three areas within the social communication and social interaction category. In the restricted, repetitive patterns of behavior, interests, or activities category, individuals need to exhibit at least two of the four symptom domains. The five children in this study were evaluated according to the diagnostic criteria of DSM-5, which include severity levels. One child had level 1 severity, one child had level 2 severity, and three had level 3 (for example, in the domain of social interaction, these levels indicate difficulties in initiating social interaction, limitations in initiating social interaction, and extremely limited initiating social interaction, respectively) (2).

The basic information of each participant was primarily obtained through in-depth interviews with researchers, which reflected the participants’ personal perspectives, emotions, and attitudes towards their children. The five participants had no prior connections. Firstly, the study employed purposive sampling rather than open recruitment. Secondly, strict confidentiality measures were applied to all research-related information. **Table 1** presents the demographic information of this study’s participants.

**Table 1:** Demographic information of caregivers and their children with autism spectrum disorder.

Pseudonym	Age/ Gender	Education	Occupation	Child name (pseudo)/Level of severity	Child age/gender	Age when first diagnosed
Fan	47/M	BA	Company employee	C1/Level 2	8/girl	3
Liu	39/M	BA	Freelancer	C3/Level 3	4/girl	3
Wang	37/M	Ph.D	Teacher	C2/Level 3	7/boy	3.5
Li	39/F	Ph.D	Teacher	C5/Level 3	4/girl	3
Zhang	37/F	MA	Senior manager in a bank	C4/Level 1	7/boy	3.5

*Participant 1*: Fan. His personality is quiet and relatively passive in conversation, and when he looks at his daughter with ASD, his eyes are full of fatherly love. He once confided that raising two children made him feel suffocated. He is willing to spend everything on his daughter’s treatment but also has to consider the material support that his elder daughter needs. The repeated daily routine makes him tired and pessimistic.

*Participant 2:* Liu. He is the primary caregiver of his only daughter, who was diagnosed with ASD about one year ago. Liu’s understanding and expression of fatherly love is to respond to all his child’s demands. Typically, he seems friendly and optimistic. However, when faced with his child’s emotional outbursts, he often complains about losing control and feeling helpless.

*Participant 3:* Wang. He states that his son was once his glory. He met all of Wang’s expectations before the child was three years old. After the child was diagnosed with ASD, frequent intervention routines became the entirety of Wang’s life. He takes his son back and forth between home and the treatment center every day. He has repeatedly expressed that he expects the intervention to be effective and eagerly hopes that his child will have the happiness and life of an ordinary child.

*Participant 4:* Li. Her child’s diagnosis forced her to become a more resilient mother. She actively seeks the best treatment plan for her child. During our interview, she repeatedly expressed her desire for her child to be like other children. Additionally, she does not hesitate to choose her child over professional career development.

*Participant 5:* Zhang. She describes her relationship with her son as “two creatures probing at each other.” While raising her son alone as a single mother, she wants to be a good mother. However, she constantly realizes her incompetence and unfamiliarity with how to express maternal love.

### 2.3 Data collection

The data were collected through semi-structured individual interviews, which consisted of several key questions concerning (1) the parents’ different emotional responses and changes after learning their children have autism, (2) how they view and understand their children’s unique behavioral patterns, (3) their expectations for their children’s future life, (4) the key skills or abilities they hope their children will possess, and (5) the impact of their children’s ASD symptoms on their life. These key questions help define the areas to be explored and allow the interviewer or interviewee to diverge and pursue an idea or response in more detail. This kind of interview format of gathering data is frequently used in therapy and healthcare as it can guide clients on subjects of discussion, which significantly helps them. In qualitative research, an interview is supposed to explore the individual’s experiences, views, beliefs, and motivations on specific matters (49). Individual semi-structured interviews with caregivers were conducted in a quiet space at a therapy center in China. The interview was conducted in Chinese (the participants’ native language) to facilitate extensive verbal sharing. Nonverbal information such as facial expressions and tone of voice were not coded systematically across transcripts, but were noted by the researcher where they appeared notable.

### 2.4 Data analysis

The interview materials were recorded and transcribed digitally using Clova Note (50). The researcher then checked their accuracy by reading the transcription and listening to the audio-recorded file. The qualitative data analysis software MAXQDA (51) was used for text data analysis.

The researchers used Moustakas’s (52) method to analyze the data. The analysis program includes the following four stages: (1) reading the individual interview transcription several times, which helped the researcher understand each individual more deeply and have a sense of wholeness about the data; (2) highlighting all meaningful statements and writing memos beside them (coding); (3) peer-debriefing by sharing original transcriptions with codes; (4) combining overlapping and repetitive codes and eliminating vague expressions; (5) categorizing similar meaningful statements and codes; (6) developing themes for the categories after reviewing categories and codes numerous times; (7) peer-debriefing by sharing original transcriptions with codes, categories, and themes; and (8) writing textual descriptions of participant experiences.

### 2.5 Triangulation

Two types of triangulations were adopted in this study.

Member checking is a way to explore the credibility of results. It is also known as participant or respondent validation. Later, data or results are sent back to participants to give them a chance to check for accuracy (53). Participants confirmed and provided feedback on the content of the interview transcription (electronic files). Upon reviewing the interview transcription, Wang requested that the researcher delete the interview content related to “family members’ relationships.” Liu requested that the researcher delete any discussions involving individuals unrelated to this study. The researcher respectfully removed all requested relevant content by the participants from the interview transcription.

In qualitative research, peer debriefing is a collaborative process of sharing and discussing results, understanding, and methods with peers. This process allows researchers to have critical dialogues, receive fresh insights, and share their understandings (54–57). This study invited two peer reviewers to review and provide feedback on the data analysis program (i.e., the export process of coding-category-theme) to ensure the objectivity and accuracy of the analysis results. The peer reviewers consistently scrutinized the researcher’s thoughts and feelings throughout the analysis. Reviewers commented on the researchers’ analysis methods and results during the peer debriefing process. They constantly posed questions like “How else can this question be expressed?” “Why do you think so?” and “Please explain your response.” These questions prompted the researchers to constantly reflect on the data, their thinking, and research methods.

### 2.6 Researcher reflections

This study could be conducted because the researcher is a dance/movement therapist. Initially, the study focused on the intervention efficacy of DMT for children with ASD, which was published in the *American Journal of Dance Therapy* (58). In the past few years, the primary investigator, Fan, provided DMT services to children with ASD. This professional role gradually provided access to caregivers in Beijing, China. With this access came an awareness of the difficulties that parents raising children with ASD in Chinese culture are confronted by, and attention was paid to their parenting and emotional experiences. Therefore, the study naturally extended to caregivers of children with ASD. To provide a more supportive environment for caregivers, an exploration of their parenting experiences is required. In collecting and analyzing data, the researcher’s roles and responsibilities are emphasized to focus, more intensely, on the caregiver’s parenting experience instead of the therapeutic effects on the child. The investigator strove to suppress the therapist’s professional identity. Prolonged engagement helped the researcher to understand the sensitivity of this study subject and its contents.

### 2.7 Ethical consideration

This study is derived from Fan’s doctoral dissertation (59), approved by the Institutional Review Board (IRB) of Jeonju University (JJIRB-230503-HR-2023-0406). The data used in this study are a portion of those not used for the author’s doctoral dissertation, which has been deemed meaningful to explore. The study was explained to all participants, and they were informed of their right to refuse or withdraw from the study without any penalties. All information was collected with the consent of the participants, and the responses were processed anonymously.

## 3 Results

This study aims to clarify the parenting experiences of Chinese parents raising a child with ASD. The qualitative data analysis of interview transcription revealed four themes, 15 categories, and 67 codes. **Table 2** presents the results of the data formation. These findings will be discussed with textual descriptions and direct quotes.

**Table 2:** Themes and categories derived from interview transcripts.

Themes (4)	Categories (15)
Theme 1: Unable to abandon the hope that the child will become “ordinary”	The hope that the child will live like other children
	The desire for total recovery
	Trying many different therapeutic interventions
	Expectations for future treatment technology
Theme 2: Child’s development goes against the expectations of their caregivers	The child’s expressions are monotonous and restricted
	The child keeps a distance and is unapproachable
	The child is unpredictable
	The child’s emotional issues are difficult to handle
Theme 3: Emotional phases in the caregiver’s responses to diagnosis	Anger, negation, and avoidance
	Sadness and fear
	Confrontation and acceptance
Theme 4: Enduring the life that was given	Caregivers’ burnout
	Caregiver’s worries
	Child-centered family life
	Chaos

Owing to space limitations, 67 codes were excluded from this table.

### 3.1 Theme 1: Unable to abandon the hope that the child will become “ordinary.”

This theme includes four categories: a. The hope that the child could live like other children, b. The desire for total recovery, c. Trying many different therapeutic interventions, and d. Expectations for future treatment technology. The first theme describes how participants struggle to give up hope that a full recovery will make their child “ordinary.”

Fan, a father, expressed:

Since entering school, my child has been able to keep up and occasionally interact with other classmates. I feel happy for my child, and this is the first step toward our success. As parents, we do not expect our child to excel in her studies. During class, as long as she can sit still, put her hands on the desk, and look at the teacher … I just hope my child can have her own life like her peers.

Li expressed similar hopes as Fan. She said,

My child is more sensitive than other children. However, in the future, as a member of society, I hope that she can express her dislike, refuse appropriately, or just remain silent. I hope she can be understood and become a part of the collective rather than expressing emotional ups and downs solely by stomping, kicking, and crying.

A sense of hope is deeply rooted in the wishes of the caregivers, which has reinforced their desire for ASD recovery. Wang shared his thoughts: “I don’t want to look back on the past two years, whether it’s good or bad … After my child was diagnosed, I had blind optimism. Maybe after the spring or summer, his symptoms will be gone like a page of a book has been flipped over so that he can finally be just like other children.” Two other caregivers also mentioned, “In the early stages of intervention, I always had a fantasy that after a few days or months of treatment, my child might show significant changes”; “I even hoped that his problems would improve after one week.”

Owing to the high expectations for the effectiveness of rehabilitation, caregivers wished that every treatment method was an “elixir.” Additionally, because of their inadequate ability to accompany their children with ASD, they placed all their hopes on treatment. Parents take their children to interventions all year without a rest. They said, “As long as we can afford it economically, we are willing to make every effort for our children.” Some interviewees have also detailed various treatments they know can treat ASD, including one-on-one, small groups, group therapy, language intervention, cognitive therapy, sensory integration, and motor synthesis. Additionally, Li has also described her “family style intervention.”

I have read about guiding children with ASD through eye contact in books. Afterward, I tried attracting my child’s attention. For example, when I discuss something with my child, his father is also willing to join us actively, but he always mechanically and rigidly commands: “Look at your father’s eyes and speak.” Now, whenever my child makes a request, he will also rigidly repeat, “Look into my dad’s eyes and speak.”

In addition to current expectations for ASD, caregivers shared their visions for the future. They hope that in the future, ASD will no longer be troublesome, an obstacle, or a problem. For example, Wang shared that:

Many young people currently prefer to be alone rather than have a partner. In the future, marriage, family, and romance may not be the necessities of life. Companion robots can replace romantic partners, voice assistants can be used for language difficulties, and one can always order takeouts when hungry. What people like my child cannot do can all be done with technology. This is what I believe.

### 3.2 Theme 2: Child’s development goes against the expectations of their caregivers

This theme comprises four categories: a. The child’s expressions are monotonous and restricted; b. The child keeps a distance and is unapproachable; c. The child is unpredictable, and d. The child’s emotional issues are difficult to handle. In this theme, the caregivers talk about the indescribable pain they experience when facing their children’s ASD symptoms and challenging behaviors.

Parents report that their children’s language deficiency and lack of interaction have hindered emotional communication and development between parents and children. Fan admitted, “She is unable to say what I hope to hear. When I ask a question, her answer is never what I want. It’s difficult for me and my child to form and develop a conversation.” Li also said, “My child doesn’t have the ability to respond to others yet. If she does not rely on external cues and support, her response is usually not responding or ignoring.”

This unbreakable emotional barrier causes the caregiver to fail repeatedly and eventually become disheartened. Liu said helplessly, “Even if I call her loudly, she always turns a deaf ear.” Fan recalled, “It’s difficult for me to have eye contact with my child, even if she’s right across from me. The kind of back-and-forth communication is too luxurious for me.” Zhang frankly stated that as a mother, she felt she had failed in her mother–son relationship with her child. “I strive to create a harmonious family atmosphere of ‘unconditional tolerance for my child,’ but my child does not feel very close to me. He does not seem to crave emotional communication with me, that is what I think.”

More often than not, their children’s unexpected behavior and emotional problems also challenge their patience and hope. “When my child is stable, she will pester me to sing and recite ancient poems,” “We can do anything together, and he will even initiate hugs … However, the child’s state is unpredictable, and perhaps in the next second, she will cry or struggle uncontrollably.” The children seem to create problems repeatedly while their caregivers must clean up their messes. As a result, they are chronically angry and on the verge of losing control.

The caregivers have buried their true wishes as they care for their children with ASD. The children behave contrary to the caregivers’ original aspirations, and their behaviors are unpredictable. However, caregivers often feel helpless and burdened by the unfair realities they face, yet they find themselves unable to vent or cry about these challenges. Additionally, they have forced themselves to pretend that nothing significant had happened, using their weak hope for the future to offset the physical and mental exhaustion brought about by their confrontations with ASD.

### 3.3 Theme 3: Emotional phases in the caregivers’ responses to diagnosis

This theme consists of three categories: a. Anger, negation, and avoidance; b. Sadness and fear; and c. Confrontation and acceptance. Parents described the personal emotional changes they experienced when facing their children’s autism. The most frequently used words were negation, avoidance, anger, sadness, and fear. Although these pessimistic feelings continue to spread, the Chinese caregivers ultimately choose to accept that their children’s autism will not go away. Fan said softly, “I do not like to mention the word ‘autism,’ even though it is a fact.” Wang’s reaction to his son is not so equable:

His perception of the world feels shrouded in a film or mist. His attention is not focused. It is only when I say, “I want to hit you,” and he feels scared that his attention returns. However, when I usually talk to him, his mind is not here, which really makes me angry.

The caregivers’ frame of mind becomes more agitated as their children grow. They reported, “I can feel it. I cannot avoid the outside world and colleagues. Social media platforms alone are filled with a variety of information. For example, colleagues scheduling weekend dinners and rejecting their invitations require reasonable reasons.”

Regardless of whether the outside world is kind or cruel, worry and sadness are the caregivers’ primary emotions. Liu said, “It is too heavy, like the sky has collapsed. Now … I can accept even if my child cannot go to college … even if she cannot get into vocational high school, I will keep raising her as long as she is happy.”

Wang said in a low voice:

Sometimes, I would quietly look at my child and get emotional. He cannot feel the world like a normal child. Ordinary people understand what friendship and love are, but my child may not feel it or cannot feel it like we do. I have too many regrets. However, Zhuangzi (c. 369 BC–c. 286 BC, a representative figure in Taoism) once said, “Only a fish knows the joy of a fish.” Are we certain that what we think is right and good for our child is always good? He has his world, only that we have not been there.

Complaining and feeling angry, depressed, and sad are useless. The caregivers’ only choice is to gather themselves and move forward. Therefore, they put aside their “vanity of comparison,” making comparisons with how other children develop and excel. They work harder and live to provide their children with a level of material wealth; They also begin to accept “fate,” as Wang said, “As God did not give me a choice, I can only choose to face it.”

### 3.4 Theme 4: Enduring the life that was given

This theme consists of four categories: a. Caregivers’ burnout, b. Caregivers’ worries, c. Child-centered family life, and d. Chaos. Theme 4 reflects the reality faced by Chinese caregivers who raise children with ASD. Even if the caregivers have tried to understand and accept ASD, the daily situations and countless tests they still have to face can drain their minds and bodies and decrease their confidence.

Wang said in a hopeless tone,

I am numb to the hardships of life. We have really reached the limit. Watching his repetitive and rigid behavior is unbearable. I cannot help but blame him and then blame myself. Sometimes, I even beat and scold him, and then I regret it. I know it is not his fault. He may have come from another planet, and what he sees and feels is different from others. He works hard to adapt to this world, but we insist on correcting him according to the rules of society. He is really in pain…

Now, he is receiving ABA treatment, with four classes per day and two classes on Saturdays. Maybe handing him over to the treatment center is me slacking off. However, watching him all day is too tiring. Now, my patience with him can last for at most two hours, and I really cannot give it anymore. So, I would rather spend money and let others help me take care of him.

ASD has become the center of life for these Chinese parents, and the more they want to get rid of its influence, the more suffocating it becomes. “This reality is testing our patience. For example, our child’s mother has watched him fall asleep and wake up for seven years.”

Fan also said,

Previously, we were worried that she would be different from other kindergarten children. Now that our child is in school, we are worried that she will be rejected by her classmates and teachers. I worry about her every day, and perhaps one day, she will be required to drop out of school.

The more the parents put in effort to protect their children, the less effective it seems. A caregiver said, “At home, our child has everything she needs. If she wants to watch cartoons, I will put them on. If she wants to eat, I will set the dishes for her.” Wang’s face showed an unsettled expression.

I am very conflicted. Is it really good for him, or does it really make him happy when I try so hard to correct him? I always want to know the answer. Our so-called interventions actually follow Western scientific rationality, which says that after something happens, we should look for the cause and effect and make changes. However, according to our Chinese thinking, we surrender to the natural way of the Tao. According to this concept, should we not conform to the child rather than intervene? We are forcing him to do things that he cannot do and does not like to do, and at the same time, if he cannot do them well, he gets scolded and beaten. This is really a contradiction.

Caregivers have to face and endure the reality that ASD cannot be completely cured and that they will be sacrificing a lot of their time as they take care of their children. “I am not myself anymore; I am no longer who I was.”

## 4 Discussion

This study explored Chinese parenting experiences of raising children with ASD by focusing on emotional experiences and their perceived roles and responsibilities.

### 4.1 Research question 1: What were the Chinese caregivers’ emotional experiences of raising their children with ASD?

The words of the interviewees showed the emotional states of pain, oppression, and entanglement of Chinese caregivers of children with ASD. The emotional responses of these caregivers are rife with contradictions and oppositions, as evidenced by both Themes 1 and 3 described in the previous section. The understanding of similar emotional states is defined as “the embrace of paradox” in Larson’s *Reforming the Means of Disability to Families: The Embrace of Paradox* (60). This phenomenon of embracing paradox is particularly prominent in our research process. For example, while denying their child’s ASD with statements such as, “I do not want to mention the word ‘autism’”; “I don’t want to look back on the past two years, whether it’s good or bad,” and “I never want my child to be labeled as ‘autistic,’ and no one has the right to define her in this way.” At the same time, they show a state of accepting ASD through responses such as “For these years, we have been repeatedly [taking the child to] intensive interventions from Monday to Saturday,” “No matter what kind of treatment it is, we will try it as long as it may be helpful for our child. I am willing to do everything for our child.”

The above paradoxical statements or viewpoints may serve as a microcosm of the tension between the traditional Chinese concept of “responsibility of passing down the family line” and the maintenance of personal reputation within a collectivist culture.

In China, descendants (children) are expected to inherit and expand the social and material conditions of the family, thereby promoting its prosperity. Additionally, there is a practical need for “raising children to prevent aging,” where parents expect their children to “be filial when they grow old” and fulfill their obligation to support the parents. This traditional concept intensifies the wish of Chinese caregivers to have a “normal child.” However, in a collectivist society, the family is viewed as a whole. Once a child’s issues are exposed, the entire family may be subjected to external criticism and judgment. Therefore, avoiding and concealing the potential impact and harm to the family’s reputation from conditions such as “autism” has become a consensus among family members, as “autism” is classified as a mental disability in China rather than a form of neurodiversity.

The researcher believes that the exposition of cultural concepts can help us understand the emotional state of Chinese caregivers. However, the driving force based on emotions is fragile and vulnerable. When the expectations of caregivers for their children to “recover” gradually fall through, when hope turns into sadness and despair, and when reality repeatedly hits the caregivers, they may emotionally break down and suffer from psychological trauma.

### 4.2 Research question 2: How do Chinese caregivers perceive their roles and responsibilities towards their children with ASD?

Regardless of being from the East or West, the importance of children to their parents is far more significant and complex than the arrival of a new life. In China, the birth of a child marks the ultimate completion of a traditional family. From birth, children are regarded by their parents as the glory and hope of the family.

Cultural beliefs also influence Chinese caregivers who raise children with ASD. Unlike ordinary families, these caregivers consciously attribute their children’s shortcomings to their mistakes. For example, they think, “This is punishment for me (as a parent)” and “I have committed sins, and therefore my child is not perfect.” The deeper the condemnation imposed by caregivers on themselves, the more excessive their guilt and compensatory behavior toward their children. Some families devote all their resources to choosing and providing treatment for their children (61); some families use all their social connections to fight for educational resources for their children (62). Concerning parenting style, some caregivers are like helicopters hovering over their children, exerting high-pressure control over everything; others are like lawnmowers, always running ahead of their children. Before the children open their mouths and start doing things, their parents deprive them of the opportunity to independently experience and develop their abilities.

The parents believe these strategies are the best ways to show love for their children during their upbringing. They do not realize that such excessive interference may hinder and limit the children’s growth and development. Moreover, the gap between the excessive caring of the parents and the actual condition of the children with ASD may accelerate the physical and mental exhaustion of the parents and lead to them experiencing the helplessness of being parents who are unable to fulfill their responsibilities.

Given the emotional challenges faced by caregivers, parents require substantial support. Seeking social support is one of the most frequently reported needs of parents of children with ASD (63); however, the lack of sufficient social support is also one of the most frequently reported sources of stress for these parents (63–65). Parents of children with ASD can obtain support through both informal and formal channels. Informal social support includes an individual’s direct social network, such as friends and family. Although studies have shown that informal support is associated with reduced parental pain (66) and improved adaptation of the family (67), Lu et al. (68) confirmed that Chinese parents receive a lower level of social support.

Formal support refers to activities conducted through organized institutions, professionals, or groups (69). Unfortunately, the government’s financial support and subsidies for children with ASD are minimal. The development of education placement, allocation of rehabilitation resources, and childcare services for children with ASD are also lacking (70). Therefore, most Chinese parents can only rely on their own family to raise their children with ASD. Under the guidance of collectivist values, the family is considered the primary source of support, with a strong emphasis on interdependence and mutual support between spouses. However, this tendency towards excessive self-reliance and familial responsibility not only increases the pressure of raising children but may also lead to detrimental cycles in caregivers’ psychological, emotional, daily life, and social interactions.

### 4.3 Limitations and future research directions

This study has some limitations. Most Chinese caregivers of children with ASD are unwilling to disclose their experiences owing to the stigma related to ASD and emotional difficulties. This reticence limited the number of respondents. Furthermore, this study was conducted in Beijing, China’s capital. This city is highly modernized. Expanding the research to other provinces and rural areas in China could reveal different experiences of caregivers of children with ASD.

Sameroff and Chandler have pointed out that all human development, including disability, is rooted in social culture and influenced by dynamic processes (71). Therefore, the attitudes and actions of caregivers are inevitably shaped and limited by their specific cultural environment. In the West, attitudes towards autism are generally more positive and open. Western caregivers generally hold optimistic beliefs that by seeking appropriate interventions and faithfully implementing them, children have the potential to achieve progress in their recovery (72, 73). In the East, especially in China, attitudes towards autism are often negative. Mostly, people do not like to discuss disability as if hoping that it would disappear if it is not seen. According to Kleinman, disability is seen as a negative presence in China, depriving individuals of their legal rights (74). Therefore, families and communities often hesitate to invest their scarce resources in children (75).

This difference in understanding autism is also reflected in the roles and responsibilities of caregivers (44, 76). In the West, “good mothers” usually strive for the best resources and related services for their children and are committed to exploring and unleashing their children’s potential. In the East, “good mothers” are more often described as psychologically enmeshed with their children, even to the extent of sacrificing their desires to cure their children’s disabilities (77).

These different perspectives on autism have a significant impact on the parenting styles of caregivers. Therefore, in order to more effectively support Chinese caregivers, cultural differences should be fully considered, and targeted caregiver support plans and propositions should be developed to improve society’s understanding and attitudes toward disability. For example, to develop an effective localized caregiver support system, prioritize key elements such as mental health interventions, stress management training, and personalized educational strategy guidance. Formulate service standards in alignment with China’s Mental Health Plan (2015–2020) (78). At the community level, implement a resource coordination plan to create a sustainable support network, utilizing community-based participatory empowerment mechanisms (79).

## 5 Conclusion

In this study, Chinese caregivers provided in-depth descriptions of their parenting experiences, especially the complex emotional changes they have experienced while raising their children. The caregivers’ emotional process of accepting their children’s disabilities and diagnosis is a topic worth exploring. Further understanding will help reveal the stages of an emotional process model for caregivers of children with ASD in a culture where child–parent relationships are perceived differently compared to in a Western culture. The researchers believe that the purpose of qualitative research is not to extract universal patterns but to gain deeper insight and understanding of the inherent nature of human experience. Therefore, when presenting the results, we strove to avoid subjective assertions and assumptions, to fully respect and truthfully reflect the feelings and experiences of the caregivers, and to re-present the caregiver’s parenting experiences and emotional states to the greatest extent possible.

This study reflects the authentic parenting dilemmas and intense emotional fluctuations experienced by Chinese caregivers of children with ASD. This study’s contribution is that it provides a real and vivid opportunity for caregivers of children with disabilities to speak out. In the Chinese context, this group of people receives very little attention, and they have no platform to voice their experiences. This study helps to reshape the public’s perception of disability and enhance the understanding of the family situations of children with disabilities in China. Additionally, it suggests that the government and society should provide institutional and emotional support for caregivers of children with ASD, promoting social understanding and acceptance.

## Data Availability

The datasets presented in this article are not readily available because data is confidential. Requests to access the datasets should be directed to Xing Fan, n1fx11go@naver.com.
